# Vitamin B12 deficiency associated with hyperbilirubinemia and cholestasis in infants

**DOI:** 10.12669/pjms.343.14564

**Published:** 2018

**Authors:** Sahin Erdol, Taner Ozgur

**Affiliations:** 1 Sahin Erdol, MD, Division of Metabolism, Uludag University Faculty of Medicine, Department of Pediatrics, 16059, Gorukle,Bursa, Turkey; 2 Taner Ozgur, MD, Division of Gastroenterology, Uludag University Faculty of Medicine, Department of Pediatrics, 16059, Gorukle, Bursa, Turkey

**Keywords:** Cholestasis, Hyperbilirubinemia, Vitamin B12 deficiency

## Abstract

**Objective:**

To study the correlation between vitamin B12 deficiency and hyperbilirubinemia and cholestasis in infants.

**Methods:**

The study group consisted of 215 infants who were tested for serum B12 and bilirubin levels out of 335 cases referred to the Centre from June 2011 to 2016 as a part of the screening program established by the Ministry of Health. The following information was obtained from the case files: demographic data; background; family history; serum vitamin B12, folate, plasma homocysteine, and urine methylmalonic acid (MMA) levels; and direct, indirect, and total bilirubin levels.

**Results:**

About 48.8 percent of cases had vitamin B12 deficiency. No significant differences were found when those cases with vitamin B12 deficiency and those without vitamin B12 deficiency were compared in terms of total, direct, or indirect bilirubin levels. Only two cases (0.9 percent) had cholestasis.

**Conclusion:**

The study suggests vitamin B12 deficiency is a common phenomenon (48.4 percent), similar to what has been suggested by other studies conducted in Turkey. Therefore, the presence of vitamin B12 deficiency in cases with cholestasis or hyperbilirubinemia may show an association. To prove the correlation between them, more studies are required.

## INTRODUCTION

It is necessary to eat food of animal origin, such as meat, milk, and eggs, to take in vitamin B12, as it cannot be synthesized in the human body. This is especially true for people having a poor socioeconomic status; because the most common reason for vitamin B12 deficiency is poverty. Infants would not be expected to suffer from vitamin B12 deficiency as long as the mother has no such deficiency, because vitamin B12 is transferred to the fetus via the placenta during pregnancy.^1^ Vitamin B12 deficiency causes many health problems including growth and developmental delays and hematological findings as vitamin B12 acts as a cofactor in DNA synthesis, methylation, neurotransmitter synthesis, and the homocysteine/methionine cycle.^2^ Vitamin B12 is essential for the maturation and proliferation of erythrocytes. In the case of vitamin B12 deficiency, erythrocytes cannot mature with consequent hemolysis and hyperbilirubinemia.^3^ Several in-vitro studies suggest that increased homocysteine resulting from vitamin B12 and folic acid deficiency may cause hemolysis.^4^ Literature search showed a few studies regarding the relation between vitamin B12 deficiency and the presence of cholestasis.

This study addressed the association between vitamin B12 deficiency and hyperbilirubinemia and cholestasis in infants referred to the Metabolism Centre as a part of the screening program established by the Ministry of Health.

## METHODS

Data for this retrospective study was obtained from the files of 215 infants tested for serum B12 and bilirubin levels out of 335 cases referred to the Division of Pediatric Metabolism and Nutrition from June 2011 to 2016. All cases except for one were born mature and referred for suspicion of phenylketonuria or biotinidase deficiency.

The following information was obtained from the case files: age at the time of referral; sex; gestational age; birth weight; mother’s age and number of pregnancies; existence of consanguineous marriage; previous diagnoses; serum vitamin B12, folate, plasma homocysteine, and urine methylmalonic acid (MMA) levels; and serum total, direct, and indirect bilirubin levels.

In total, 200 pg/ml was accepted as the threshold value for a vitamin B12 deficiency diagnosis.^5^ A laboratory diagnosis of cholestasis was established: when the direct bilirubin level was greater than 20 percent of the total bilirubin level if the total bilirubin level was greater than 5 mg/dL or when the direct bilirubin level was higher than 1 mg/dL if the total bilirubin level was lower than 5 mg/dL.^6^

All laboratory data were obtained from the tests conducted at the biochemistry laboratory of the Centre. The following devices were used to test the following: Architect i2000 (Abbott Diagnostics, USA) using the chemiluminescence micro particle immunoassay (CMIA) method for serum vitamin B12 and folic acid levels, Immulite 2000 (Siemens Diagnostics, NJ, USA) using the chemiluminescence immunoassay (CLIA) method for plasma homocysteine levels, Perkin Elmer (Clarus 680 GC-600TMS, USA) using the gas chromatography mass spectrometer method for urine organic acids and methylmalonic acid levels, and Architect c16000 (Abbott Diagnostics, USA) using the spectrophotometric end-point method for serum total and direct bilirubin levels.

All procedures followed were in accordance with the ethical standards on human experimentation (institutional and national) and with the Helsinki Declaration of 1975, as revised in 2000. Written informed consent was obtained from the parents of the patient prior to inclusion in the study.

### Statistical analysis

Whether the study data was consistent with a normal distribution was evaluated with the Shapiro-Wilk test. Variables consistent with a normal distribution and independent samples were compared by t-test, and descriptive statistics were given as the average ± standard deviation. Data inconsistent with a normal distribution were compared using the Mann–Whitney U test and descriptive statistics were given as the median (minimum–maximum). Pearson’s chi-square test was used for an intergroup comparison of categorical variables, and Spearman’s correlation analysis was used to evaluate the relationship between variables. For statistical analyses, the package program IBM SPSS Statistics 2.1 was used. The results obtained were interpreted as significant if p<0.05.

## RESULTS

Baby boys and baby girls accounted for 55.3 percent (n=119) and 44.7 percent (n=96) of the 215 cases included in the study, respectively. The average referral age, birth week, and birth weight were 25.3±1.48 days, 39±0.12 weeks, and 3.171±36.6gr respectively. No inherited metabolic diseases that would cause hyperbilirubinemia or cholestasis were detected. The cases did not have any disease that may lead to pathological hyperbilirubinemia and they had not received phototherapy.

The average serum vitamin B12 level was found to be 259±13.2 pg/mL, where 48.8 percent of the infants were vitamin B12 deficient taken 200 pg/ml as cut-off for deficiency. The serum folate level for all 195 cases, where the serum folate level could be tested, was within the normal range (average=13.8±3.1 ng/mL; minimum=4, maximum=21) and no deficiency was found.

No significant difference was found between the group with vitamin B12 deficiency and the group without vitamin B12 deficiency in terms of age, sex, and serum total bilirubin (p=0.300), direct bilirubin (p=0.294), and indirect bilirubin (p=0.529) levels. In contrast, there was a significant difference in terms of birth week and birth weight (Table-I). The birth week and birth weight for the group with vitamin B12 deficiency were 39.4±1.34 weeks and 3.256±520.8 gr respectively, and these values were 38.6±2.14 weeks and 3.091±532.7 gr for the group without vitamin B12 deficiency, respectively. The greater average gestational age and birth weight among the infants with vitamin B12 deficiency might be due to the fact that the fetus receives vitamin B12 from the expectant mother with active transport; the longer the gestation period, the more apparent the deficiency in the fetus becomes for the infants whose expectant mothers have vitamin B12 deficiency.

**Table-I:** Demographic and clinical characteristics of cases with and without vitamin B12 deficiency.

	Cases with vitamin B12 deficiency	Cases without vitamin B12 deficiency	P
Gender (M/F)	42/63	54/56	0.180
Age (day)	28.1±29.1	22.6±10.4	0.060
Gestational age (week)	39.4±1.34	38.6±2.14	0.006
Birth weight (gr)	3256±520.8	3091±532.7	0.024
Serum vitamin B12 level (pg/mL)	132.7±43.9	380.4±204.7	0.001
Serum total bilirubin level (mg/dL)	5.23±3.94	4.63±3.91	0.300
Serum indirect bilirubin level (mg/dL)	4.66±3.88	4.28±3.98	0.529
Serum direct bilirubin level (mg/dL)	0.43±0.18	0.48±0.43	0.294

p<0.05 is meaningful

No significant difference was detected in terms of the serum total (p=0.598), direct (p=0.735), and indirect (p=0.856) bilirubin levels, probably because vitamin B12 deficiency manifests as anemia when serum B12 level is below 100 pg/mL.^7^

No significant correlation was found between increased homocysteine, an important laboratory indicator of vitamin B12 deficiency, and increased serum total (p=0.793), direct (p=0.713), and indirect (p=0.747) bilirubin levels. A plasma homocysteine level of higher than 10 µmol/L was considered significant in terms of vitamin B12 deficiency.^8^ Therefore, serum total (p=0.415), direct (p=0.811), and indirect (p=0.254) bilirubin levels were compared between the cases whose homocysteine level was greater or less than 10 µmol/L, and no significant difference was found.

No significant correlation was found between urine methylmalonic acid levels—another important laboratory indicator of vitamin B12 deficiency—and the serum total (p=0.252), direct (p=0.952), and indirect (p=0.243) bilirubin levels (Spearman’s correlation analysis). As an increased urine methylmalonic acid level (normal: 0.3–1.9 mmol/mol creatinine) is significant in terms of vitamin B12 deficiency, the serum total (p=0.919), direct (p=0.451), and indirect (p=0.839) bilirubin levels were compared between the cases whose urine methylmalonic acid level was higher or lower than 1.9 mmol/mol creatinine, and no significant difference was found. Only two (0.9 percent) of 215 cases included in the study had cholestasis: one case with and the other without vitamin B12 deficiency.

## DISCUSSION

Only a few studies with small number of patients demonstrate the correlation between vitamin B12 deficiency and hyperbilirubinemia in the literature. It was found that a considerable portion of the cases referred to the Metabolism Division due to cholestasis and hyperbilirubinemia suffered from vitamin B12 deficiency. Hence, it was necessary to conduct a study including a higher number of cases to prove the correlation.

Hyperbilirubinemia emerges when there is an imbalance between the production and the elimination of bilirubin. Fetal hemoglobin produced during the intrauterine period is catabolized rapidly leading to increased production of bilirubin which is not eliminated as fast as it is produced in the newborn period. This phenomenon manifests itself as hyperbilirubinemia. In some circumstances, such as ABO and Rh group incompatibility, hypothyroidism, glucose-6-phosphate dehydrogenase deficiency, or urinary tract infection, the bilirubin level may reach pathological levels and result in severe sequelae such as kernicterus.^9^ Almost all the cases included in the study are in the neonatal period and none had pathological jaundice.

Vitamin B12 and folate metabolism is a crucial factor for DNA synthesis. Deficiency of these vitamins leads to megaloblastic anemia due to ineffective erythropoiesis. There is no definitive data about whether vitamin B12 deficiency causes hyperbilirubinemia and its mechanism. It is suggested that ineffective erythropoiesis may cause immature erythrocyte formation increasing hemolysis and indirect hyperbilirubinemia.^10^

The review of the literature showed that there are case studies suggesting vitamin B12 deficiency may cause hyperbilirubinemia. For instance, Dasari S et al.^11^ reported a patient aged 41 with hyperbilirubinemia who was diagnosed with vitamin B12 deficiency and his hyperbilirubinemia was eliminated following vitamin B12 treatment. In addition, Eroglu N et al.^10^ compared 20 cases with indirect hyperbilirubinemia of above greater than 5 mg/dL with 20 cases whose indirect bilirubin was below 5 mg/dL and found significant serum vitamin B12 deficiency in the former group. This is the only case control study identified in the literature.

Our study includes the highest number of cases in the literature to the best of the authors’ knowledge. In total, 105 (48.8 %) cases with vitamin B12 deficiency and 110 (51.2 %) cases without vitamin B12 deficiency are compared in terms of total (p = 0.300), direct (p = 0.294), and indirect bilirubin (p = 0.529) levels and no significant difference was found.

The tests for plasma homocysteine and urine methylmalonic acid levels have high sensitivity and specificity in determining the sufficiency of the serum vitamin B12 level for intracellular metabolism. The level of methylmalonic acid in the blood and urine and the level of homocysteine in the plasma increase due to the reduced activity of the methylmalonyl-CoA mutase enzyme and methionine synthase enzymes in vitamin B12 deficiency.^12^ The levels of plasma homocysteine and methylmalonic acid in the urine and the levels of total, direct, and indirect bilirubin were compared, and once again, there was no significant difference. Ventura P et al.^4^ have suggested that increased homocysteine resulting from vitamin B12 and folic acid deficiency may cause hemolysis based on an in vitro study. In our study, however, no significant difference was found when the cases were categorized into those with a homocysteine level above and below 10µmol/L. In addition, the serum total (p=0.415), direct (p=0.811), and indirect (p=0.254) bilirubin levels were not different compared with respect to the plasma homocysteine level taken 10 µmol/L as cut-off as proposed to be significant in terms of vitamin B12 deficiency.^8^

Cholestasis is defined as a decrease in bile flow. In the case of cholestasis, bilirubin, cholesterol, and bile salts normally excreted into bile cannot be excreted adequately and are retained in the tissues due to a decrease in bile flow. Cholestatic jaundice develops with increased direct bilirubin and is usually pathological. Cholestatic jaundice should be diagnosed and treated urgently. It is reported that galactosemia, tyrosinemia, bile acid metabolism disorders, and alpha-1 antitrypsin deficiency may cause cholestasis, and there is no report of cholestasis associated with vitamin B12 deficiency.^6^ In our study, we found only two cases (0.9 percent) with cholestasis: one case with and the other without vitamin B12 deficiency.

Vitamin B12 deficiency, known to be more frequent in societies with a poor socioeconomic condition, is more common in several regions of Turkey compared to developed countries. For instance, 81.6 percent of mothers and 42 percent of their newborn babies suffer from vitamin B12 deficiency according to a study conducted by Onal H et al.^13^ in Istanbul exploring serum B12 levels in 250 expectant mothers and their newborn babies. However, the percentage of adults suffering from vitamin B12 deficiency was found to be 2.6 and 3.4 percent in Switzerland and Spain, respectively.^14^

## CONCLUSION

This study suggests vitamin B12 deficiency is a common phenomenon (48.4 percent), similar to what has been suggested by other studies conducted in Turkey. Therefore, the presence of vitamin B12 deficiency in cases with cholestasis or hyperbilirubinemia may show an association. Vitamin B12 deficiency should be analyzed in cases with pathological jaundice with an unknown etiology. To prove the correlation, more studies are required.

### Author`s Contribution

**SE, TO** conceived, designed, did statistical analysis & editing of manuscript.

**SE, TO** did data collection and manuscript writing.

**SE** did review and final approval of manuscript.
